# Immune checkpoint inhibitors induced autoimmune hemolytic anemia in patients with malignant cancer

**DOI:** 10.1093/oncolo/oyag395

**Published:** 2026-10-09

**Authors:** Aoxi Liang, Yuanqiang Wu, Shengchi Chen, Jisheng Li, Yue Pan, Yixing Luo, Yue Zeng, Shijiao Yao, Shuxing Wang, Chunhong Hu, Xianling Liu, Fang Wu

**Affiliations:** Department of Oncology, The Second Xiangya Hospital, Central South University, Changsha 410011, China; Department of Oncology, The Second Xiangya Hospital, Central South University, Changsha 410011, China; Department of Oncology, Nanping First Hospital Affiliated to Fujian Medical University, Nanping 353000, China; Department of Medical Oncology, Qilu Hospital of Shandong University, Jinan 250012, China; Department of Thoracic Surgery, The First Affiliated Hospital of Guangzhou Medical University, Guangzhou 510120, China; Department of Oncology, The First Affiliated Hospital of Guangzhou Medical University, Guangzhou 510120, China; Xiangya School of Medicine, Central South University, Changsha 410013, China; Department of Oncology, The Second Xiangya Hospital, Central South University, Changsha 410011, China; Department of Oncology, The Second Xiangya Hospital, Central South University, Changsha 410011, China; Department of Oncology, The Second Xiangya Hospital, Central South University, Changsha 410011, China; Xiangya School of Medicine, Central South University, Changsha 410013, China; Department of Oncology, The Second Xiangya Hospital, Central South University, Changsha 410011, China; Hunan Cancer Mega-Data Intelligent Application and Engineering Research Centre, Changsha 410011, China; Department of Oncology, The Second Xiangya Hospital, Central South University, Changsha 410011, China; Department of Oncology, Guilin Hospital of the Second Xiangya Hospital, Central South University, Guilin 541100, China; Department of Oncology, The Second Xiangya Hospital, Central South University, Changsha 410011, China; Hunan Cancer Mega-Data Intelligent Application and Engineering Research Centre, Changsha 410011, China; Hunan Key Laboratory of Tumor Models and Individualized Medicine, The Second Xiangya Hospital, Central South University, Changsha 410011, China; Hunan Key Laboratory of Early Diagnosis and Precision Therapy in Lung Cancer, The Second Xiangya Hospital, Central South University, Changsha 410011, China; FuRong Laboratory, Changsha 410078, China; Changsha Thoracic Cancer Prevention and Treatment Technology Innovation Center, Changsha 410011, China

**Keywords:** autoimmune hemolytic anemia, immune checkpoint inhibitors, PD-1, irAE, corticosteroids, anemia

## Abstract

**Background:**

Autoimmune hemolytic anemia (AIHA) is a rare but potentially life-threatening hematologic immune-related adverse event (irAE) associated with immune checkpoint inhibitors (ICIs). This study reports 3 new cases of ICI-induced AIHA from three medical centers in China and places them in the context of existing literature.

**Methods:**

Three patients with AIHA following ICI therapy were identified from The Second Xiangya Hospital, Qilu Hospital, and Nanping First Hospital. A PubMed search up to June 1, 2026, was conducted to collect previously reported cases with sufficient clinical details.

**Results:**

A total of 51 cases were analyzed, including three new patients and 48 from published reports. The mean age was 65.2 years, and 47.1% were male. The most common tumor types were melanoma (49.0%) and non-small cell lung cancer (27.5%). Most cases were associated with anti–PD-1 agents, particularly pembrolizumab and nivolumab, and AIHA typically developed early during ICI therapy. Corticosteroids were the primary treatment, with 92.2% achieving hematologic recovery or improvement. Four deaths (7.8%) were attributed to severe hemolysis. All three newly identified patients had pre-existing anemia before ICI initiation.

**Conclusions:**

ICI-induced AIHA usually occurs early during ICI therapy and responds favorably to corticosteroid-based treatment. Pre-existing anemia in our cases should be regarded as a hypothesis-generating observation rather than evidence of a specific risk factor, underscoring the need for early hemolysis work-up and close monitoring when anemia worsens after ICI exposure.

## Background

Immune checkpoint inhibitors (ICIs) have transformed cancer therapy but can cause immune-related adverse events (irAEs) involving various organs. Hematologic irAEs are rare but may result in life-threatening complications such as autoimmune hemolytic anemia (AIHA).^1^ ICI-induced AIHA is characterized by Coombs-positive hemolysis mediated by autoantibodies and T-cell dysregulation following PD-1/PD-L1 or CTLA-4 blockade.^2^ Since its first description in 2016, well-documented cases remain limited, and clinical awareness remains insufficient.^3^ Early recognition and timely immunosuppressive therapy are essential to prevent severe anemia or fatal outcomes. Here, we review published cases and summarize additional patients from our institutions to better define clinical features and management implications.

## Methods

Three cases of AIHA following ICI therapy were identified from The Second Xiangya Hospital, Qilu Hospital, and Nanping First Hospital. A PubMed search was updated to June 1, 2026, to identify additional published cases. Only individually reported cases with sufficient clinical details were included, including cancer type, ICI agent, temporal relationship with ICI exposure, evidence of hemolysis and/or AIHA, treatment, and outcome. Duplicate reports, aggregated pharmacovigilance data without individual-level details, and cases in which AIHA could not be distinguished from other causes of anemia were excluded. The original publications corresponding to each previously reported case are provided in Table S1.

## Results

Instances of ICI-induced AIHA are rare. We identified 51 cases, including three new patients from our institutions and 48 cases retrieved from published literature after a comprehensive PubMed search. The mean age was 65.2 years (range, 18–85), and 47.1% were male. The most common tumor types were melanoma (49.0%) and non-small cell lung cancer (27.5%), followed by colorectal cancer (5.9%) and gastric adenocarcinoma (3.9%).

Most cases were associated with anti–PD-1 therapy, most frequently pembrolizumab or nivolumab, while fewer cases involved anti–PD-L1, CTLA-4, or other PD-1 inhibitors. AIHA developed after a median of two treatment cycles, with most cases occurring within the early treatment period. Laboratory tests revealed positive direct antiglobulin tests for IgG and/or C3 in 56.9% of patients, while 13.7% were DAT-negative and 21.6% had unreported DAT results. Based on hemoglobin levels, 82.3% of patients experienced grade ≥3 anemia, indicating that ICI-induced AIHA was often clinically severe.

ICIs were withheld in most cases, and all patients received glucocorticoids as first-line therapy. Additional treatments included intravenous immunoglobulin (11.8%), rituximab (7.8%), azathioprine (2.0%), and cyclophosphamide (2.0%) for selected or steroid-refractory cases. With these treatments, 92.2% achieved hematologic recovery or improvement, while four deaths (7.8%) were attributed to severe hemolysis. Among patients who underwent ICI rechallenge (29.4%), most tolerated it without recurrence of AIHA.

Overall, ICI-induced AIHA typically occurred early during ICI therapy and responded favorably to corticosteroid-based management, emphasizing the importance of timely recognition and intervention.

## Discussion

ICIs have achieved remarkable success across a wide range of malignancies, yet their expanding use has led to increasing awareness of irAEs, including rare hematologic disorders such as AIHA.^1^ Although the reported incidence of ICI-induced AIHA is less than 1%, the condition can be severe and even life-threatening if not promptly recognized.^4^

In our analysis of 51 cases, ICI-induced AIHA occurred predominantly during the early treatment phase, with a median onset after two treatment cycles (Tables 1 and 2) This pattern is consistent with pharmacovigilance data suggesting that hematologic irAEs tend to develop shortly after immune activation.^5^ The majority of affected patients were treated for melanoma or non–small cell lung cancer, which are also common tumor types treated with ICIs. Most cases were associated with anti–PD-1 agents, particularly pembrolizumab and nivolumab. However, this predominance should be interpreted cautiously, as it may reflect broader clinical use, tumor indications, treatment exposure, and reporting patterns rather than a true biologic difference in AIHA risk among ICI classes.

**Table 1.** Reported cases of ICI-induced AIHA with detailed clinical information.

Age	Gender	Diagnosis	ICIs	LDH/U/L	Hemoglobin/g/dL			Ret%	DAT mediator	Treatment	Outcome	ICIs Re-challenge
					Baseline	Nadir	**Δ**					
**62**	M	NSCLC	Pembro × 1	289	8.7	4.5	4.2	3.49	IgG, C3	MP	Recover	No
**63**	F	Intrahepatic CHOL	Cam × 2	1395	10.6	5.1	5.5	–	Negative	MP	Recover	No
**55**	F	Gastric adenocarcinoma	Nivo × 1	3288	9.7	8.5	1.2	–	–	MP	Recover	No
**52**	F	Melanoma	Pembro × 3	1266	12.5	6.3	6.2	0.1	IgG	GSs, IVIG	Recover	No
**55**	M	Melanoma	Pembro × 1	399	10.4	6.3	4.1	0.3	IgG, C3	Pred	Recover	Pembro
**67**	M	Melanoma	Pembro × 8	NR	15.4	6.6	8.8		NR	MP	Recover	No
**68**	M	Melanoma	Pembro × 12	530	13.6	8.5	5.1	4.7	IgG	Pred	Recover	Nivo
**79**	F	Melanoma	Pembro × 1	235	–	5.0	–	–	IgG, C3	GCs	Recover	No
**81**	F	Melanoma	Pembro × 2ys	1176	9.1	3.9	5.2	8.9	IgG, C3	MP, Pred	Recover	No
**85**	M	Melanoma	Pembro × 2	885	12.8	8.2	4.6	3.3	Negative	Pred, Rituximab	Recover	No
**85**	F	Melanoma	Pembro × 10	1151	12.9	6.4	6.5	6.4	IgG	Pred, IVIG, azathioprine	Recover	No
**71**	F	NSCLC	Pembro × 4	623	10.1	5.5	4.6	0.3	Negative	Dex	Recover	Pembro
**73**	M	NSCLC	Pembro × 13	1615	11.0	7.4	3.6	NR	NR	Pred	Recover	No
**78**	M	NSCLC	Pembro × 1	492	11.7	6.0	5.7	7.8	NR	Pred	Recover	NR
**69**	F	CRC	Pembro × 1	716	11.7	9.4	2.3	4.3	NR	Pred	Recover	No
**52**	M	NSCLC	Nivo × 24	383	13.4	5.9	7.5	18.9	IgG	MP	Expired	No
**59**	F	NSCLC	Nivo × 12	505	11.9	6	5.9	16.8	IgG, C3	Pred, IVIG, Rituximab	Recover	No
**63**	F	NSCLC	Nivo × 4	976	11.7	4	7.7	14.8	IgG	MP, Pred	Recover	No
**60**	M	NSCLC	Nivo × 21	335	8-9	4.3	–	17	IgG	MP, Pred	Recover	Nivo
**70**	M	NSCLC	Nivo × 2	330	10.5	5.5	5.0	NR	C3	Pred	Expired	No
**78**	M	NSCLC	Nivo × 39	648	11-13	8.6	–	17.0	IgG	Pred, rituximab	Recover	No
**75**	F	Hodgkin’s lymphoma	Nivo × 2	409	12.3	6.4	5.9	NR	IgG	Pred	Recover	Nivo
**82**	M	Cutaneous SCC	Nivo × 8	NR	NR	NR	–	NR	IgG, C3	Pred	Recover	NR
**85**	M	Melanoma	Nivo × 5	682	>10	6.4	–	7.9	IgG	Pred	Recover	No
**33**	F	AML	Nivo × 1	791	9.3	7.2	2.1	4.9	Negative	MP, Pred	Recover	No
**47**	M	Melanoma	Ipi × 1	522	9.5	6.2	3.3	3.2	NR	Dex	Recover	Ipi
**68**	F	Melanoma	Ipi × 3	580	NR	6.0	–	0.2	NR	MP	Recover	NR
**71**	M	Melanoma	Ipi × 4	NR	NR	NR	–	NR	IgG, C3	Pred	Recover	NR
**18**	M	Melanoma	Ipi + Nivo × 4	784	12.4	6.2	6.2	<0.5	Negative	MP, Pred	Recover	Ipi + Nivo
**43**	F	Melanoma	(Ipi + Nivo) × 2	1406	>10	5.6	>4.4	6.5	IgG, C3	MP, Rituximab	Recover	Ipi + Nivo
**48**	M	Melanoma	(Ipi + Nivo) × 3	770	7.2	3.5	3.7	11.1	IgG, C3	MP, Pred	Recover	No
**67**	F	Melanoma	(Ipi + Nivo) × 4	426	13.2	8.7	4.5	8.8	IgG	Pred	Recover	Nivo
**67**	M	Melanoma	(Ipi + Nivo) × 2	1574	13.7	6.2	7.5	8.5	Negative	Dex, Pred	Recover	Ipi + Nivo
**64**	F	Melanoma	Ipi × 7 + Nivo × 15	326	11.7	7.6	4.1	NR	IgG, C3	Pred	Recover	Pembro
**75**	M	Melanoma	Pembro × 1	925	15.1	7.7	7.4	NR	Negative	Pred	Recover	No
**29**	F	Melanoma	(Ipi + Nivo) × 3	492	12.4	6.1	6.3	NR	NR	MP	Recover	No
**67**	M	Melanoma	Pembro × 1	331	12.5	8.5	4	NR	IgG	Pred	Recover	Pembro
**71**	M	NSCLC	Pembro × 1	674	12	3.4	8.6	4.42	IgG, C3	MP	Recover	No
**65**	F	NSCLC	Atezo	NR	11	6	5	NR	IgG, C3	Pred	Recover	No
**47**	F	NSCLC	Pembro × 2	396	–	6.1	–	NR	C3	Pred	Expired	No
**56**	F	TNBC	Atezo	396	12.1	7.5	4.6	12	NR	Pred	Recover	No
**51**	F	CRC	Pembro × 1ys	486	10.0	4.3	5.7	10.3	NR	MP	Recover	No
**84**	F	ESCC	Sintilimab × 2	308	–	5.5	–	15.2	IgG, C3	MP, IVIG	Recover	No
**69**	M	NSCLC	Pembro × 1	1214	12.3	4.5	7.8	23.7	IgG, C3	MP, IVIG	Recover	No
**84**	F	GAC	Sintilimab × 1; Cad/Ivo	487	9.9	4.7	5.2	4.5	IgG	GCs	Expired	Cad/Ivo
**52**	F	Melanoma	Pembro × 1	1573	NR	2.9	NR	31.4	Positive	MP, IVIG, CTX	Recover	Pembro
**82**	F	CRC	Tori × 1	856	8.2	4.5	3.7	9.0	Positive	Pred	Recover	No
**66**	F	Endometrial cancer	Pembro × 3	420	10.0	4.9	5.1	NR	IgG	Pred	Recover	Dost
**74**	M	Melanoma	Ipi × 1	1348	8.5	4.06	4.44	NR	Positive	GCs	Recover	No
**84**	M	Melanoma	Nivo × 7 mo	600	NR	6.6	NR	NR	NR	GCs	Recover	No
**78**	F	Melanoma	Pembro × 2	263	12.0	11.4	0.6	NR	IgM cold autoantibody	Pred	Improved	No

The corresponding original reports for previously published cases are provided in Table S1. Abbreviations: AIHA, autoimmune hemolytic anemia; AML, acute myelocytic leukemia; Atezo, Atezolizumab; Cad, cadonilimab; Cam, Camrelizumab; CHOL, cholangiocarcinoma; CRC: colorectal cancer; CTX, cyclophosphamide; DAT, direct antiglobulin test; Dex, dexamethasone; Dost, dostarlimab; ESCC, esophageal squamous cell carcinoma; F, female; GAC, gastric adenocarcinoma; GCs, glucocorticoids; ICIs, immune checkpoint inhibitors; Ipi, ipilimumab; IVIG, intravenous immunoglobulin; Ivo, ivonescimab; LDH: lactate dehydrogenase; M, male; MP, methylprednisolone; Nivo, nivolumab; NR, not reported; NSCLC, non-small cell lung cancer; Pembro, pembrolizumab; Pred, prednisone; Ref., reference; Ret%, percentage of reticulocyte; SCC, squamous cell carcinoma; Sin, sintilimab; ys, years.

**Table 2.** Characteristics of reported patients with ICI-induced AIHA.

Characteristics	ICIs induced AIHA (*n* = 51)
**Sex, *n* (%)**	
** Female**	27 (52.9)
** Male**	24 (47.1)
**Age in years, average (range)**	65.2 (18-85)
** Female**	63.7 (29-85)
** Male**	66.9 (18-85)
**Cancer type, *n* (%)**	
** Melanoma**	25 (49.0)
** NSCLC**	14 (27.5)
** Colorectal cancer**	3 (5.9)
** Gastric adenocarcinoma**	2 (3.9)
** Lymphoma**	1 (2.0)
** Esophageal Squamous Cell Carcinoma**	1 (2.0)
** Cutaneous squamous cell carcinoma**	1 (2.0)
** Acute myelocytic leukemia**	1 (2.0)
** Triple-negative breast cancer**	1 (2.0)
** Intrahepatic cholangiocarcinoma**	1 (2.0)
** Endometrial cancer**	1 (2.0)
**Immunotherapy agent(s), *n* (%)**	
** Pembrolizumab**	22 (43.1)
** Nivolumab**	12 (23.5)
** Ipilimumab** + **Nivolumab**	7 (13.7)
** Ipilimumab**	4 (7.8)
** Atezolizumab**	2 (3.9)
** Sintilimab**	2 (3.9)
** Camrelizumab**	1 (2.0)
** Toripalimab**	1 (2.0)
**ICIs cycles before onset**	
** Median (range)**	2 (1-39)
**AIHA Grade**	
** Grade 1**	0
** Grade 2**	7 (13.7)
** Grade 3**	22 (43.1)
** Grade 4**	20 (39.2)
** Not reported**	2 (3.9)
**DAT mediator, *n* (%)**	
** IgG, C3**	14 (27.5)
** IgG**	13 (25.5)
** C3**	2 (3.9)
** IgM cold autoantibody**	1 (2.0)
** Positive, unspecified**	3 (5.9)
** NR**	11 (21.6)
** Negative**	7 (13.7)
**Treatment, *n* (%)**	
** GCs (dexamethasone, prednisone, methylprednisolone)**	51 (100)
** Intravenous immunoglobulin**	6 (11.8)
** Rituximab**	4 (7.8)
** Azathioprine**	1 (2.0)
** Cyclophosphamide**	1 (2.0)
**Recovery from ICIs induced AIHA, *n* (%)**	
** Yes**	47 (92.2)
** No**	4 (7.8)
**Re-challenge with ICIs, *n* (%)**	
** Yes**	15 (29.4)
** No**	32 (62.7)
** NR**	4 (7.8)

Abbreviations: AIHA, autoimmune hemolytic anemia; DAT, direct antiglobulin test; GCs: glucocorticoids; ICIs, immune checkpoint inhibitors.

Corticosteroids remain the cornerstone of therapy and were effective in most patients, while rituximab, intravenous immunoglobulin, or other immunosuppressive agents were used in selected steroid-refractory or severe cases.^6^ This treatment pattern is also consistent with recent reviews of hematologic irAEs, which recommend glucocorticoids as first-line therapy and rituximab or other immunosuppressive agents for refractory immune-mediated cytopenias.^7^ Despite the high proportion of grade ≥3 anemia, 92.2% of patients achieved hematologic recovery or improvement. However, four deaths were attributed to severe hemolysis, indicating that ICI-induced AIHA can be rapidly progressive and potentially fatal. ICI rechallenge may be feasible in selected patients, but should be individualized according to AIHA severity, hematologic recovery, alternative anticancer options, and expected oncologic benefit.^8–10^

Of particular note, all 3 newly identified patients in our cohort had preexisting anemia before ICI initiation. This observation should be regarded as hypothesis-generating rather than evidence that baseline anemia is a specific risk factor for ICI-induced AIHA. Anemia is common in patients with cancer and is often multifactorial; therefore, baseline anemia may mainly complicate the early recognition of hemolysis. In clinical practice, unexplained, rapidly worsening, or hemolysis-associated anemia after ICI exposure should prompt early hemolysis work-up, including reticulocyte count, LDH, indirect bilirubin, haptoglobin, and direct antiglobulin testing. This study is limited by its reliance on case reports and case series with potential publication bias and incomplete reporting, so the findings should be interpreted descriptively rather than as evidence of causal risk factors.

## Conclusion

ICI-induced AIHA is a rare but potentially life-threatening irAE that often occurs early during ICI therapy. Most patients respond favorably to corticosteroid-based treatment when recognized promptly. The observation of preexisting anemia in our newly identified cases should be regarded as hypothesis-generating rather than evidence of a specific risk factor. In patients with baseline anemia, unexplained or rapidly worsening anemia after ICI exposure should prompt early hemolysis work-up and close hematologic monitoring. Early recognition and timely intervention are essential to improve outcomes and ensure the safe use of ICIs in clinical practice.

## Supplementary Material

oyag395_Supplementary_Data

## Data Availability

The data supporting the findings of this study are available from the corresponding author upon reasonable request.

## Abbreviations

ICIs: immune checkpoint inhibitors
PD-1: programmed cell death 1
PD-L1: programmed death ligand-1
irAEs: immune-related adverse events
AIHA: autoimmune hemolytic anemia
NSCLC: non-small cell lung cancer
LDH: lactate dehydrogenase
DAT: direct antiglobulin test
IVIG: intravenous immunoglobulin

## References

[1] Kennedy LB , Salama AKS. A review of cancer immunotherapy toxicity. CA Cancer J Clin. 2020;70:86-104. 10.3322/caac.21596 31944278

[2] Leaf RK , Ferreri C, Rangachari D, et al Clinical and laboratory features of autoimmune hemolytic anemia associated with immune checkpoint inhibitors. Am J Hematol. 2019;94:563-574. 10.1002/ajh.25448 PMC9552038 30790338

[3] Delanoy N , Michot J-M, Comont T, et al Haematological immune-related adverse events induced by anti-PD-1 or anti-PD-L1 immunotherapy: a descriptive observational study. Lancet Haematol. 2019;6:e48-e57. 10.1016/S2352-3026(18)30175-3 30528137

[4] Poto R , Troiani T, Criscuolo G, et al Holistic approach to immune checkpoint inhibitor-related adverse events. Front Immunol. 2022;13:804597. 10.3389/fimmu.2022.804597 PMC9005797 35432346

[5] Urwyler P , Earnshaw I, Bermudez M, et al Mechanisms of checkpoint inhibition-induced adverse events. Clin Exp Immunol. 2020;200:141-154. 10.1111/cei.13421 PMC7160658 31989585

[6] Brahmer JR , Lacchetti C, Schneider BJ, et al Management of Immune-Related adverse events in patients treated with immune checkpoint inhibitor therapy: American Society of Clinical Oncology Clinical Practice guideline. J Clin Oncol. 2018;36:1714-1768. 10.1200/JCO.2017.77.6385 PMC6481621 29442540

[7] Janson M , Cherifi F, Bardet H, et al Hematological immune-related adverse events associated with checkpoint inhibitors. Cancer Treat Rev. 2026;145:103121. 10.1016/j.ctrv.2026.103121 41905276

[8] Inno A , Roviello G, Ghidini A, et al Rechallenge of immune checkpoint inhibitors: a systematic review and meta-analysis. Crit Rev Oncol Hematol. 2021;165:103434. 10.1016/j.critrevonc.2021.103434 34343657

[9] Zhao Q , Zhang J, Xu L, et al Safety and efficacy of the rechallenge of immune checkpoint inhibitors after immune-related adverse events in patients with cancer: a systemic review and meta-analysis. Front Immunol. 2021;12:730320. 10.3389/fimmu.2021.730320 PMC8503641 34646270

[10] Liu S-J , Yan L-J, Wang H-C, et al Safety, efficacy, and survival outcomes of immune checkpoint inhibitors rechallenge in patients with cancer: a systematic review and meta-analysis. Oncologist. 2024;29:e1425-e1434. 10.1093/oncolo/oyae134 PMC11546642 38940446

