# Supplementary material for: Immune checkpoint inhibitors induced autoimmune hemolytic anemia in patients with malignant cancer
Source: Oncologist. 2026 Oct 9;31(11):oyag395. doi: 10.1093/oncolo/oyag395 (PMC13674544; doi:10.1093/oncolo/oyag395)
Supplement: Supplementary file 1 [file oyag395_supplementary_data.docx]

**Immune checkpoint inhibitors induced autoimmune haemolytic anemia in patients with malignant cancer**

Table S1. Original reports of previously published cases of ICI-induced AIHA included in the present analysis.

| **Nub** | **Age** | **Gender** | **Diagnosis** | **ICIs** | **Ref.** |
| --- | --- | --- | --- | --- | --- |
| 1 | 62 | M | NSCLC | Pembro×1 | Present study |
| 2 | 63 | F | Intrahepatic CHOL | Cam×2 | Present study |
| 3 | 55 | F | Gastric adenocarcinoma | Nivo×1 | Present study |
| 4 | 52 | F | Melanoma | Pembro×3 | [1] |
| 5 | 55 | M | Melanoma | Pembro×1 | [2] |
| 6 | 67 | M | Melanoma | Pembro×8 | [3] |
| 7 | 68 | M | Melanoma | Pembro×12 | [2] |
| 8 | 79 | F | Melanoma | Pembro×1 | [4] |
| 9 | 81 | F | Melanoma | Pembro×2ys | [5] |
| 10 | 85 | M | Melanoma | Pembro×2 | [2] |
| 11 | 85 | F | Melanoma | Pembro×10 | [2] |
| 12 | 71 | F | NSCLC | Pembro×4 | [2] |
| 13 | 73 | M | NSCLC | Pembro×13 | [6] |
| 14 | 78 | M | NSCLC | Pembro×1 | [7] |
| 15 | 69 | F | CRC | Pembro×1 | [2] |
| 16 | 52 | M | NSCLC | Nivo×24 | [8] |
| 17 | 59 | F | NSCLC | Nivo×12 | [2] |
| 18 | 63 | F | NSCLC | Nivo×4 | [2] |
| 19 | 60 | M | NSCLC | Nivo×21 | [9] |
| 20 | 70 | M | NSCLC | Nivo×2 | [10] |
| 21 | 78 | M | NSCLC | Nivo×39 | [11] |
| 22 | 75 | F | Hodgkin’s lymphoma | Nivo×2 | [12] |
| 23 | 82 | M | Cutaneous SCC | Nivo×8 | [13] |
| 24 | 85 | M | Melanoma | Nivo×5 | [14] |
| 25 | 33 | F | AML | Nivo×1 | [2] |
| 26 | 47 | M | Melanoma | Ipi×1 | [2] |
| 27 | 68 | F | Melanoma | Ipi×3 | [15] |
| 28 | 71 | M | Melanoma | Ipi×4 | [16] |
| 29 | 18 | M | Melanoma | Ipi+Nivo×4 | [2] |
| 30 | 43 | F | Melanoma | (Ipi+Nivo)×2 | [17] |
| 31 | 48 | M | Melanoma | (Ipi+Nivo)×3 | [2] |
| 32 | 67 | F | Melanoma | (Ipi+ Nivo)×4 | [2] |
| 33 | 67 | M | Melanoma | (Ipi+ Nivo)×2 | [2] |
| 34 | 64 | F | Melanoma | Ipi×7+ Nivo×15 | [18] |
| 35 | 75 | M | Melanoma | Pembro×1 | [18] |
| 36 | 29 | F | Melanoma | (Ipi+Nivo)×3 | [19] |
| 37 | 67 | M | Melanoma | Pembro×1 | [20] |
| 38 | 71 | M | NSCLC | Pembro×1 | [21] |
| 39 | 65 | F | NSCLC | Atezo | [22] |
| 40 | 47 | F | NSCLC | Pembro×2 | [23] |
| 41 | 56 | F | TNBC | Atezo | [24] |
| 42 | 51 | F | CRC | Pembro×1ys | [25] |
| 43 | 84 | F | ESCC | Sintilimab×2 | [26] |
| 44 | 69 | M | NSCLC | Pembro×1 | [27] |
| 45 | 84 | F | GAC | Sintilimab×1; Cad/Ivo | [28] |
| 46 | 52 | F | Melanoma | Pembro×1 | [29] |
| 47 | 82 | F | CRC | Tori×1 | [30] |
| 48 | 66 | F | Endometrial cancer | Pembro×3 | [31] |
| 49 | 74 | M | Melanoma | Ipi×1 | [32] |
| 50 | 84 | M | Melanoma | Nivo×7 mo | [32] |
| 51 | 78 | F | Melanoma | Pembro×2 | [32] |

# References

1.	Nair, R., S. Gheith, and S.G. Nair, *Immunotherapy-Associated Hemolytic Anemia with Pure Red-Cell Aplasia.* N Engl J Med, 2016. **374**(11): p. 1096-7.

2.	Leaf, R.K., et al., *Clinical and laboratory features of autoimmune hemolytic anemia associated with immune checkpoint inhibitors.* Am J Hematol, 2019. **94**(5): p. 563-574.

3.	Ni, D., F. AlZahrani, and M. Smylie, *AIHA and Pancytopenia as Complications of Pembrolizumab Therapy for Metastatic Melanoma: A Case Report.* Case Rep Oncol, 2019. **12**(2): p. 456-465.

4.	Robilliard, B., et al., *A case of pembrolizumab-induced autoimmune haemolytic anaemia with polymyalgia rheumatica.* Eur J Cancer, 2018. **103**: p. 281-283.

5.	Williams, H. and R. Aitchison, *Pembrolizumab-induced autoimmune haemolytic anaemia and cholangitis.* BMJ Case Rep, 2019. **12**(12).

6.	Johnstone, P. and O. Khan, *Pembrolizumab-associated autoimmune haemolytic anaemia.* BMJ Case Rep, 2019. **12**(10).

7.	Okawa, S., et al., *Pembrolizumab-induced Autoimmune Hemolytic Anemia and Hemophagocytic Lymphohistiocytosis in Non-small Cell Lung Cancer.* Intern Med, 2019. **58**(5): p. 699-702.

8.	Tanios, G.E., P.B. Doley, and R. Munker, *Autoimmune hemolytic anemia associated with the use of immune checkpoint inhibitors for cancer: 68 cases from the Food and Drug Administration database and review.* Eur J Haematol, 2019. **102**(2): p. 157-162.

9.	Algaze, S.D., et al., *Autoimmune haemolytic anaemia in a patient with advanced lung adenocarcinoma and chronic lymphocytic leukaemia receiving nivolumab and intravenous immunoglobulin.* BMJ Case Rep, 2018. **2018**.

10.	Palla, A.R., et al., *Autoimmune Hemolytic Anemia as a Complication of Nivolumab Therapy.* Case Rep Oncol, 2016. **9**(3): p. 691-697.

11.	Shaikh, H., et al., *A case of autoimmune haemolytic anaemia after 39 cycles of nivolumab.* BMJ Case Rep, 2018. **2018**.

12.	Tardy, M.P., et al., *Autoimmune hemolytic anemia after nivolumab treatment in Hodgkin lymphoma responsive to immunosuppressive treatment. A case report.* Hematol Oncol, 2017. **35**(4): p. 875-877.

13.	Schwab, K.S., et al., *Development of Hemolytic Anemia in a Nivolumab-Treated Patient with Refractory Metastatic Squamous Cell Skin Cancer and Chronic Lymphatic Leukemia.* Case Rep Oncol, 2016. **9**(2): p. 373-8.

14.	Kong, B.Y., et al., *Autoimmune hemolytic anemia induced by anti-PD-1 therapy in metastatic melanoma.* Melanoma Res, 2016. **26**(2): p. 202-4.

15.	Simeone, E., et al., *Serious haematological toxicity during and after ipilimumab treatment: a case series.* J Med Case Rep, 2014. **8**: p. 240.

16.	Ramos, B., G. Gastal, and R.K. Rovere, *An Autoimmune Haemolytic Anaemia Secondary to Ipilimumab Treatment.* Klin Onkol, 2017. **30**(2): p. 128-130.

17.	Khan, U., et al., *Immunotherapy-associated autoimmune hemolytic anemia.* J Immunother Cancer, 2017. **5**: p. 15.

18.	Fetter, T., et al., *Severe autoimmune hemolytic anemia following immunotherapy with checkpoint inhibitors in two patients with metastatic melanoma: a case report.* Front Immunol, 2024. **15**: p. 1342845.

19.	Olson, D.J., et al., *A case of dual-mechanism immune-related anaemia in a patient with metastatic melanoma treated with nivolumab and ipilimumab.* J Immunother Cancer, 2020. **8**(1).

20.	Hwang, S.R., et al., *Recurrent checkpoint inhibitor-induced warm agglutinin autoimmune hemolytic anemia in a patient with metastatic melanoma.* Am J Hematol, 2020. **95**(7): p. E169-E171.

21.	Zhang, X., et al., *A 71-year-old male with a life-threatening recurrence of hemolytic anemia, thrombocytopenia, and acute kidney injury after pembrolizumab therapy: a case report.* BMC Geriatr, 2023. **23**(1): p. 478.

22.	Endo, Y., et al., *Marked, Lasting Disease Regression and Concomitantly Induced Autoimmune Hemolytic Anemia and Hemophagocytic Lymphohistiocytosis in a Patient With Lung Adenocarcinoma and Autoantibodies Receiving Atezolizumab Plus Chemotherapy: A Case Report.* JTO Clin Res Rep, 2022. **3**(1): p. 100263.

23.	Jain, N.A., et al., *Association Between RBC Antigen Allo-Antibodies and Immune-Related Adverse Events During Immune Checkpoint Inhibitor Treatment for Advanced Cancers.* Cancer Manag Res, 2020. **12**: p. 11743-11749.

24.	Younce, C.M., J.M. Lawton, and D.R. Patel, *Atezolizumab-induced hemolytic anemia - A case report.* J Oncol Pharm Pract, 2021. **27**(4): p. 1026-1028.

25.	Pei, X., et al., *Efficacy and challenges of anti-PD1 in MSI-H mCRC: a case report on concurrent infections and ir-AIHA.* Front Oncol, 2024. **14**: p. 1407312.

26.	Qiu, Z., et al., *Immunotherapy-associated autoimmune hemolytic anemia induced by anti-PD-1 therapy in esophageal cancer: A case report and literature review.* Medicine (Baltimore), 2025. **104**(15): p. e42174.

27.	Adeoye, F.W., et al., *Early-Onset Autoimmune Hemolytic Anemia from Pembrolizumab in a Patient with Metastatic Lung Cancer: A Case Report.* Am J Case Rep, 2025. **26**: p. e946630.

28.	Yu, M., et al., *Case Report: Challenges in immunotherapy for the elderly: a case of refractory ICI-induced AIHA and thrombocytopenia in advanced gastric cancer.* Front Immunol, 2025. **16**: p. 1679817.

29.	Ye, Q., et al., *Severe immune-related autoimmune hemolytic anemia induced by pembrolizumab: a case report with novel immunosuppressive strategy.* Front Immunol, 2026. **17**: p. 1811425.

30.	Ma, X., et al., *Severe immunotherapy-related autoimmune hemolytic anemia induced by toripalimab in a patient with deficient mismatch repair colorectal cancer: a case report and literature review.* Front Med (Lausanne), 2025. **12**: p. 1678754.

31.	Khamis, Z., et al., *Successful Dostarlimab Rechallenge Following Pembrolizumab-Induced Autoimmune Hemolytic Anemia: A Case Report.* Clin Case Rep, 2025. **13**(8): p. e70776.

32.	Falcone, R., et al., *Hematologic immune-related adverse events in skin cancer patients treated with immune checkpoint inhibitors: a case series.* Front Pharmacol, 2025. **16**: p. 1717727.
